# Linking Zetaproteobacterial diversity and substratum type in iron-rich microbial mats from the Lucky Strike hydrothermal field (EMSO-Azores observatory)

**DOI:** 10.1128/aem.02041-23

**Published:** 2024-01-09

**Authors:** Aina Astorch-Cardona, Giliane P. Odin, Valérie Chavagnac, Alain Dolla, Hélène Gaussier, Céline Rommevaux

**Affiliations:** 1 Aix-Marseille University, Université de Toulon, CNRS, IRD, MIO, Marseille, France; 2 Laboratoire Géomatériaux et Environnement, Université Gustave Eiffel, Marne-la-Vallée, France; 3 Géosciences Environnement Toulouse, CNRS UMR 5563 (CNRS/UPS/IRD/CNES), Université de Toulouse, Observatoire Midi-Pyrénées, Toulouse, France; Colorado School of Mines, Golden, Colorado, USA

**Keywords:** Zetaproteobacteria, ZetaOTU, iron-rich microbial mats, iron oxides, Lucky Strike hydrothermal Field, EMSO-Azores observatory

## Abstract

Zetaproteobacteria have been reported in different marine and terrestrial environments all over the globe. They play an essential role in marine iron-rich microbial mats, as one of their autotrophic primary producers, oxidizing Fe(II) and producing Fe-oxyhydroxides with different morphologies. Here, we study and compare the Zetaproteobacterial communities of iron-rich microbial mats from six different sites of the Lucky Strike Hydrothermal Field through the use of the Zetaproteobacterial operational taxonomic unit (ZetaOTU) classification. We report for the first time the Zetaproteobacterial core microbiome of these iron-rich microbial mats, which is composed of four ZetaOTUs that are cosmopolitan and essential for the development of the mats. The study of the presence and abundance of different ZetaOTUs among sites reveals two clusters, which are related to the lithology and permeability of the substratum on which they develop. The Zetaproteobacterial communities of cluster 1 are characteristic of poorly permeable substrata, with little evidence of diffuse venting, while those of cluster 2 develop on hydrothermal slabs or deposits that allow the percolation and outflow of diffuse hydrothermal fluids. In addition, two NewZetaOTUs 1 and 2 were identified, which could be characteristic of anthropic iron and unsedimented basalt, respectively. We also report significant correlations between the abundance of certain ZetaOTUs and that of iron oxide morphologies, indicating that their formation could be taxonomically and/or environmentally driven. We identified a new morphology of Fe(III)-oxyhydroxides that we named “corals.” Overall, our work contributes to the knowledge of the biogeography of this bacterial class by providing additional data from the Atlantic Ocean, a lesser-studied ocean in terms of Zetaproteobacterial diversity.

**IMPORTANCE:**

Up until now, Zetaproteobacterial diversity studies have revealed possible links between Zetaproteobacteria taxa, habitats, and niches. Here, we report for the first time the Zetaproteobacterial core microbiome of iron-rich mats from the Lucky Strike Hydrothermal Field (LSHF), as well as two new Zetaproteobacterial operational taxonomic units (NewZetaOTUs) that could be substratum specific. We highlight that the substratum on which iron-rich microbial mats develop, especially because of its permeability to diffuse hydrothermal venting, has an influence on their Zetaproteobacterial communities. Moreover, our work adds to the knowledge of the biogeography of this bacterial class by providing additional data from the hydrothermal vent sites along the Mid-Atlantic Ridge. In addition to the already described iron oxide morphologies, we identify in our iron-rich mats a new morphology that we named corals. Finally, we argue for significant correlations between the relative abundance of certain ZetaOTUs and that of iron oxide morphologies, contributing to the understanding of the drivers of iron oxide production in iron-oxidizing bacteria.

## INTRODUCTION

Iron oxidation in oceans was assumed to be abiotic until the finding of iron-oxidizing bacteria in marine environments in 1995 (1). The first strains of marine iron oxidizers, *Mariprofundus ferrooxydans* PV-1 and JV-1 belonging to the Zetaproteobacteria class (2), were isolated from samples collected from the Kamaʻehuakanaloa Seamount (previously known as Lōʻihi, as it will be referred to throughout the text) near Hawaii (2, 3). Since then, 17 strains of Zetaproteobacteria have been isolated from different marine environments (2–13).

While members of this class have been mainly documented at active hydrothermal sites, their occurrence is increasingly reported in other marine environments that are more or less rich in iron (from the coast to the deep ocean) (14), in terrestrial ecosystems (9, 15), and even in the gut of the shrimp *Rimicaris exoculata* (16). ZetaHunter (17) allows a classification based on 16S rRNA gene sequences in previously defined Zetaproteobacterial operational taxonomic units (ZetaOTUs) (17). Prior analyses of Zetaproteobacterial diversity using this tool have revealed that some ZetaOTUs could be cosmopolitan, while others are enriched in specific environments, ascribing the diversity of Zetaproteobacteria to their niches and habitats (18).

All representatives of this class are characterized by the use of dissolved Fe(II) (dFe) or zero-valent iron as their main energy source (2). Such biotic Fe(II) oxidation provides very little energy (19). Under oxic conditions and at neutral pH, it competes with abiotic Fe(II) oxidation, which is faster (20). This, together with their need for oxygen, explains why Zetaproteobacteria are usually found in oxic-anoxic transitional zones (18), such as at hydrothermal fields, where reduced, heated hydrothermal fluids mix with cold, oxygenated seawater, creating redox gradients that allow the development of unique ecosystems (2, 21). As a result, orange-colored iron-rich microbial mats develop near fissures from which diffuse hydrothermal fluids discharge. These materials are composed of loosely aggregated Fe(III)-oxyhydroxides and organic polymers formed by iron oxidizers (22), which are considered to be one of the primary producers within these mats (14, 18, 23). Indeed, Zetaproteobacteria produce different morphologies of Fe(III)-oxyhydroxides (i.e., stalks, sheaths, and Y-structures), which are interpreted as being associated with differing niches in terms of dissolved Fe(II) and O_2_ gradients (24). Thus far, the majority of iron-rich mats have been described as being mainly composed of either stalks or sheaths (24).

Zetaproteobacterial diversity at hydrothermal sites has mostly been studied in the Pacific Ocean (4, 25–29). In contrast, fewer sites have been investigated on the Arctic Mid-Ocean Ridge (AMOR) (30, 31) and the Mid-Atlantic Ridge (MAR) (23). Recently, analyses of iron-rich mats have been performed at the Lucky Strike Hydrothermal Field (LSHF) on the MAR (32). In a former paper, we analyzed how the spatial and temporal variations in environmental conditions influence the microbial communities in two iron-rich mats from the LSHF (32) without investigating their Zetaproteobacterial diversity, which is the focus of the present study. Here, we explore the influence of substratum lithology and permeability on the Zetaproteobacterial diversity of six iron-rich mats from LSHF. Through scanning electron microscopy (SEM) analyses of iron oxides, we report a new morphology of Fe(III)-oxyhydroxide that we named “corals,” as well as significant correlations between iron oxide abundances and that of specific ZetaOTUs.

## MATERIALS AND METHODS

### Site and sample description

**Fig 1:**
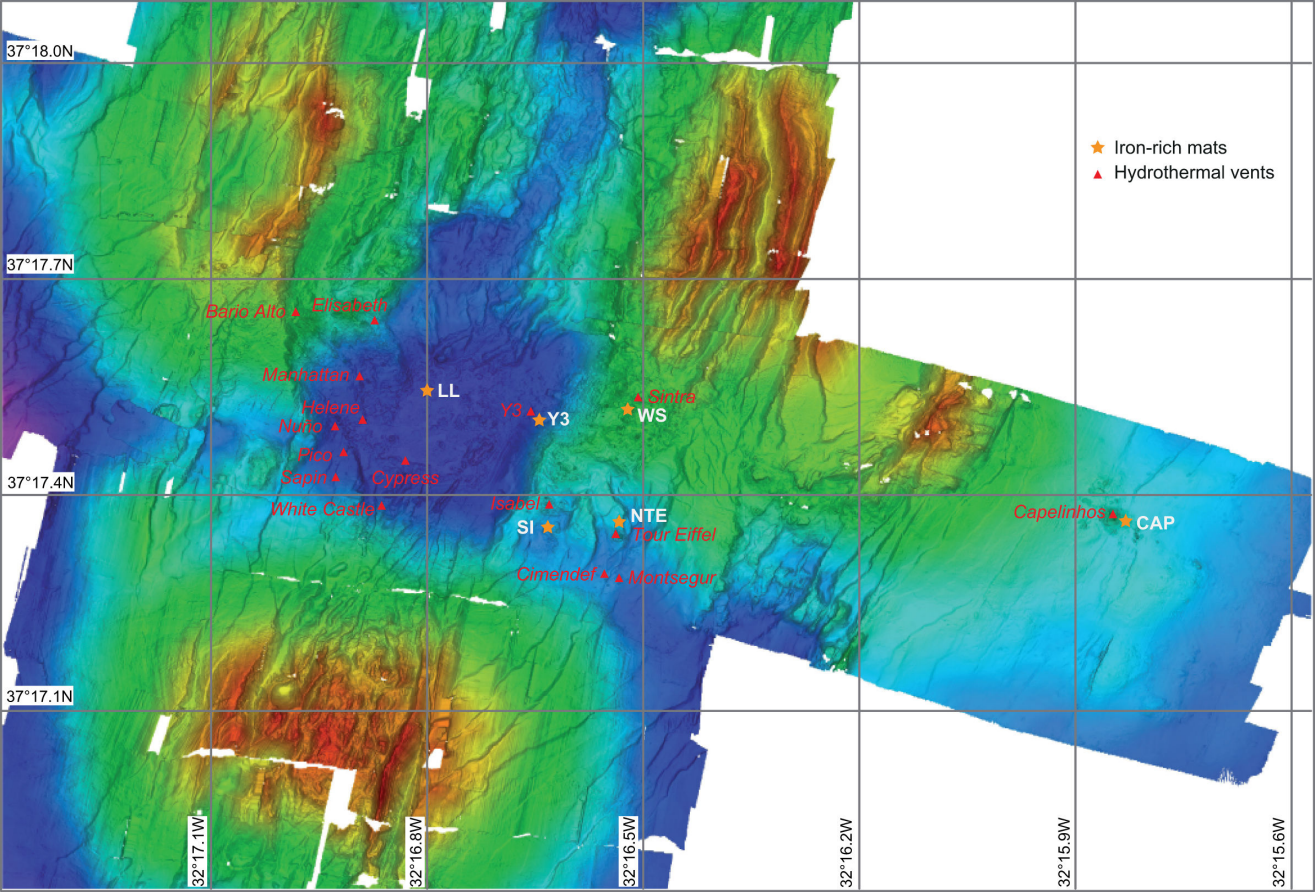
Bathymetric map [based on data from reference (33) and modified from reference (32)], representing the location of the different active sites (red triangles) and the sampled iron-rich microbial mats (orange stars) of the LSHF.

**Fig 2:**
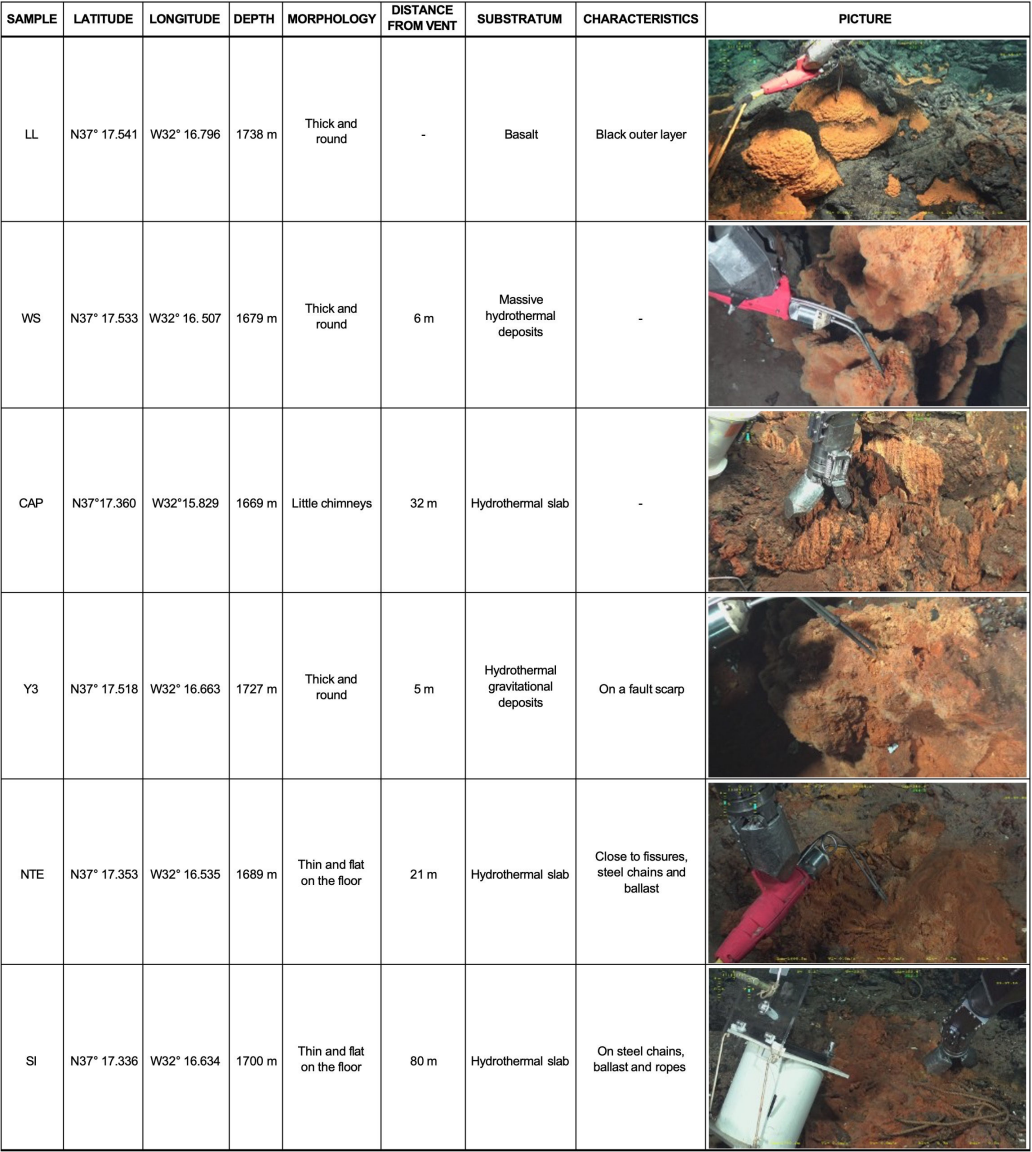
Location, depth, macroscopic characteristics, type of substratum, and pictures of the six iron-rich microbial mats sampled for this study.

The LSHF (N37°17/W32°17) is located ~400 km southwest of the Azores archipelago at an active 65-km-long ridge segment of the MAR (33). It comprises 20–30 active sites (34) that are distributed around an ~300-m-diameter fossil lava lake located in a depression surrounded by three volcanic cones, except for the Capelinhos site, which is situated ~1.5 km eastward from the historical LSHF (33, 35) (Fig. 1). For this study, iron-rich microbial mats were collected from the Lava Lake (LL), West Sintra (WS), Capelinhos (CAP), Y3 (Y3), North Tour Eiffel (NTE), and South Isabel (SI) sites (Fig. 1 and 2; Supplementary Material). At LL, iron-rich microbial mats develop directly on basalt in the middle of the fossil lava lake, where no hydrothermal activity has been detected thus far (33, 35). At WS, they form on oxidized massive sulfide blocks (36) to the west side of the Sintra edifice. CAP mats develop on a hydrothermal sulfide mound hosting a crack network with diffuse vents (37, 38). Y3 mats are located on hydrothermal gravity-waste deposits associated with an extended network of cracks releasing diffuse fluids located at the base of a steep scarp fault (34). At NTE, the mats form on an indurated hydrothermal slab to the north of the Tour Eiffel edifice, crossed by a large network of fissures discharging diffuse fluids (21, 39) and close to waste steel chains (± 1 m away). SI mats develop on top of steel chains, ballasts, and ropes on an indurated hydrothermal slab associated with diffuse fluid venting south of the Isabel site (40).

### Sample collection

Iron-rich microbial mats were yearly collected at the six sites during the annual MoMARSAT EMSO-Azores Observatory maintenance cruises (41–45) onboard the R.V. *L'Atalante* or *Pourquoi Pas?* from 2017 to 2021 at the CAP and NTE sites and in 2020 and 2021 at the other sites. *In situ* sample collection was performed with the human-operated vehicle (HOV) *Nautile* or the remotely operated vehicle (ROV) *Victor 6000*. The protocols and onboard sample processing of the iron-rich mats are described in reference (32).

### DNA extraction and 16S rRNA gene sequence analyses

Total genomic DNA was extracted in triplicate from each microbial mat sample using the FastDNA SPIN Kit for Soil (MPBiomedicals, Irvine, CA, USA) and the DNeasy PowerSoil Kit (QIAGEN, Hilden, Germany) as described in reference (32). For each sample, the six extraction products (triplicates for both kits) were pooled together prior to sequencing.

16S rRNA gene sequencing was performed on MR DNA (Shallowater, TX, USA) using Illumina MiSeq technology for three different sequencing runs (Table S1). The 341F and 785R primers targeting the V3-V4 hypervariable regions of the 16S rRNA gene for bacteria (46) were used to perform a PCR using the HotStarTaq Plus Master Mix Kit (QIAGEN, Germantown, MD, USA) (Supplementary Material). The samples were multiplexed using unique dual indices, pooled together in equal proportions, and purified using calibrated Agencourt AMPure XP magnetic beads. An Illumina DNA library was produced from PCR products, and sequencing was performed on a MiSeq instrument following the manufacturer’s instructions.

Data analysis was performed in the R environment (47) unless otherwise specified. Raw Illumina sequences were demultiplexed using the FASTQ processor free software (MR DNA) and were treated with the DADA2 pipeline (48). Reads from each run were treated independently until the chimera identification step, as described in reference (32). Trimming parameters were set as follows: trimLeft = 0 and trimRight = 30, 40, or 50 depending on the run. A unique amplicon sequence variant (ASV) table was obtained by combining the ASV tables of each run (Table S1).

A taxonomic assignment was performed using the SILVA 138.1 database (49). ASVs corresponding to another kingdom other than bacteria were removed from the data set, and subsampling down to the lowest sequencing depth was performed to obtain the same amount of reads for all the samples for comparison. Unless otherwise specified, further sequence treatment was performed with the phyloseq package (50), and graphics were produced using the ggplot2 package (51).

16S rRNA gene sequences corresponding to Zetaproteobacteria were aligned using the SINA Aligner (v1.2.11) (52). ZetaHunter (17) was used to classify Zetaproteobacterial sequences in previously defined ZetaOTUs with 97% identity. Unless otherwise specified, the read counts’ rounded average of each ZetaOTU at each site was used for the study. We used the Basic Local Alignment Search Tool (53) for calculating the sequence similarity of the most representative ZetaOTU sequences in our data set.

### Real-time quantitative PCR

Real-time quantitative PCR (qPCR) was performed on the same DNA pools to get a quantification of the abundance of Zetaproteobacteria vs bacteria in the samples. qPCR was performed at the Plateforme Transcriptomique de l’Institut de Microbiologie de la Méditerranée (IMM, Marseille, France) on a CFX96 Real-Time PCR System (Bio-Rad, Hercules, CA, USA) with the primers Bac1369F and Prok1492R for bacteria (54) and Zeta672F and Zeta837R for Zetaproteobacteria (54, 55) (Supplementary Material). The percentage of Zetaproteobacterial 16S rRNA gene copies was calculated relative to the number of bacterial 16S rRNA gene copies in each pool (Table S2).

### Scanning electron microscopy and image analysis

Iron oxides from microbial mat samples of each site were imaged by scanning electron microscopy on a ZEISS Supra 55 VP Field Emission Scanning Electron Microscope at the Ecce Terra platform (OSU-INSU, Sorbonne Université, France) in both secondary electron mode and backscattering mode to acquire high-resolution images (Supplementary Material). To ensure the results, mats from both 2020 and 2021 were analyzed. Chemical analysis was performed on a TESCAN Clara SEM equipped with a dual EDS Bruker at the PtME Platform (MHNH, France) in secondary electron mode (Supplementary Material).

To obtain statistical data on iron oxide morphologies, a ×400 image taken in backscattering electron mode was selected for image analysis using the ImageJ software (56). Each image was then subdivided into three 900 × 600 pixel areas (Fig. S1), where the different morphologies were enumerated, except for spherical oxides (usually referred to as amorphous oxides), which were studied in a qualitative way. For each iron oxide morphology, counts from the three areas of each image were summed and normalized by sample (Table S3).

## RESULTS

### Site-by-site comparison

The percentage of retained 16S rRNA gene sequencing reads after treatment with the DADA2 pipeline varied between 17.5% and 77.3% (Table S1), with samples from the 2021 run yielding the lowest percentages of retained reads. The rarefaction curves revealed that the sequencing effort was strong enough to assess the bacterial diversity in most of the samples (Fig. S2). Data from the bacterial and Zetaproteobacterial beta diversities, studied through a non-metric multidimensional scaling (NMDS) analysis (Fig. S3A and B, respectively), showed that almost all the samples collected at the same site clustered together, regardless of the year. A significant difference was observed between the communities at each site, as demonstrated by the 0.001 *P*-value (PERMANOVA) obtained for both the bacterial and Zetaproteobacterial beta diversities. This confirmed that the microbial communities of iron-rich mats from the LSHF remain broadly stable over time, except when geological events take place (32), and allowed us to perform a site-by-site comparison of both bacteria and Zetaproteobacteria.

### Bacterial diversity at the LSHF

**Fig 3:**
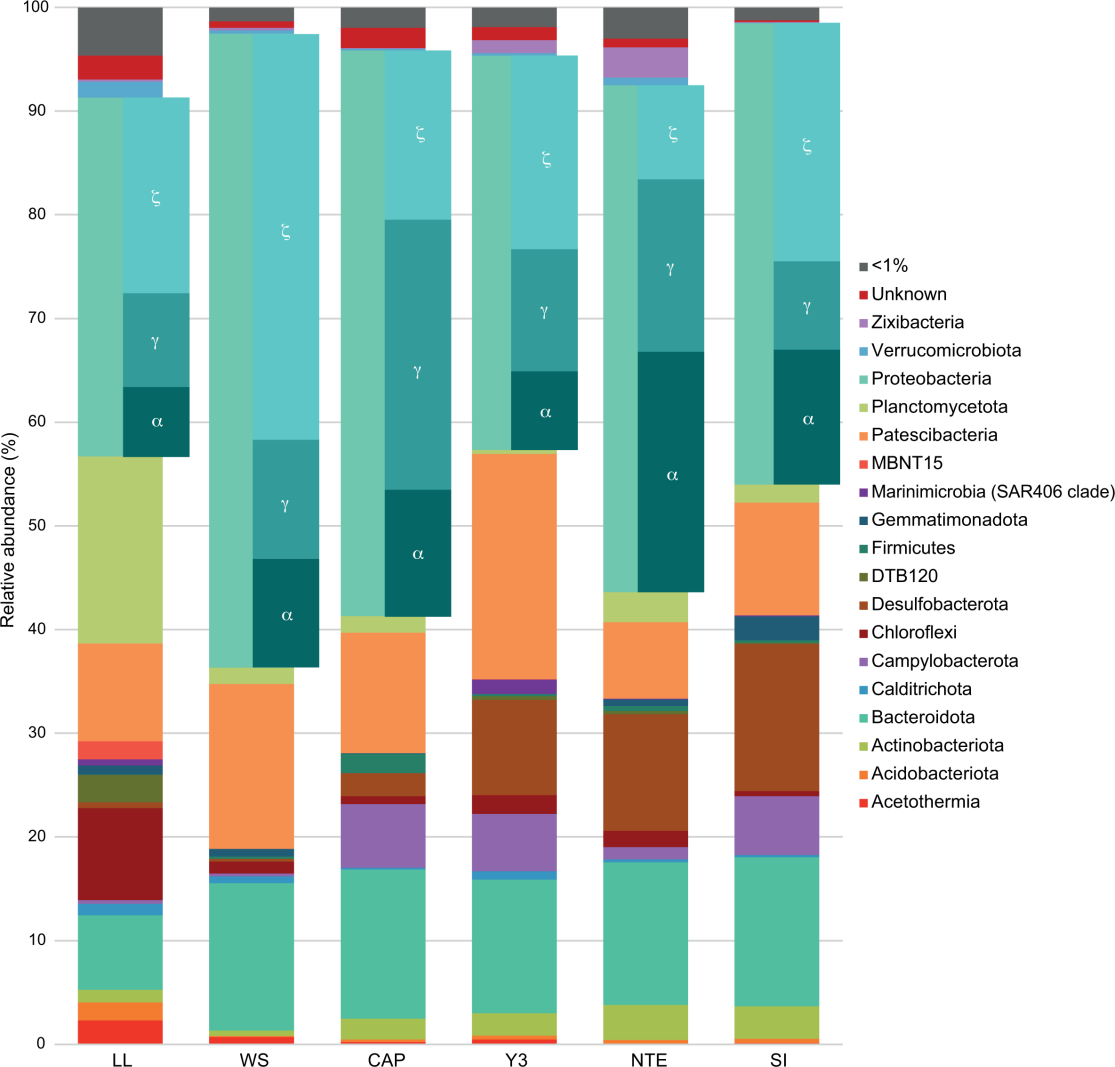
Bar plot representing the relative abundance of >1% abundant bacterial phyla in the six iron-rich mat samples.

In accordance with previous findings at CAP and NTE (32), the bacterial communities of the other four iron-rich mats were also dominated by Proteobacteria (Fig. 3; Table S4). Within this phylum, Zetaproteobacteria was the most abundant class at LL, WS, Y3, and SI, while Gammaproteobacteria and Alphaproteobacteria dominated CAP and NTE, respectively. The Proteobacteria phylum was followed by the Patescibacteria and/or Bacteroidota phyla at all sites except for LL, where Planctomycetota was the second most abundant phylum. LL and WS presented a very low abundance of both the Campylobacterota and Desulfobacterota phyla, while at CAP, Y3, NTE, and SI, these phyla had higher incidences. Finally, LL presented as well the highest abundances of members of the Chloroflexi phylum.

### Zetaproteobacterial diversity at the LSHF

**Fig 4:**
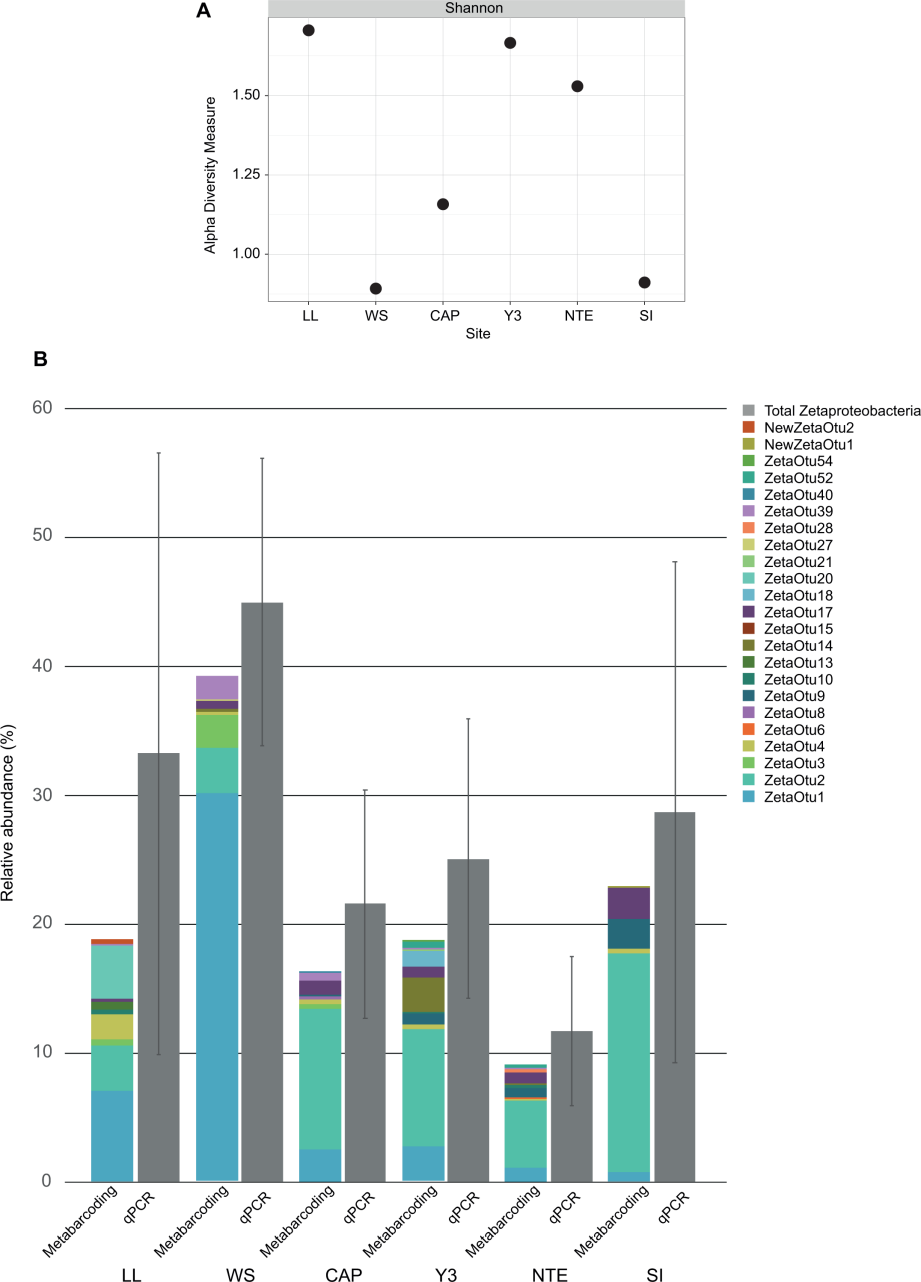
Shannon diversity index of the ZetaOTUs calculated at each site (**A**) and bar plot representing both the relative abundance (%) of Zetaproteobacteria determined by qPCR and the relative abundance (%) of each ZetaOTU at each site (**B**). Zetaproteobacterial abundances as determined by qPCR and metabarcoding were consistent with one another.

**Fig 5:**
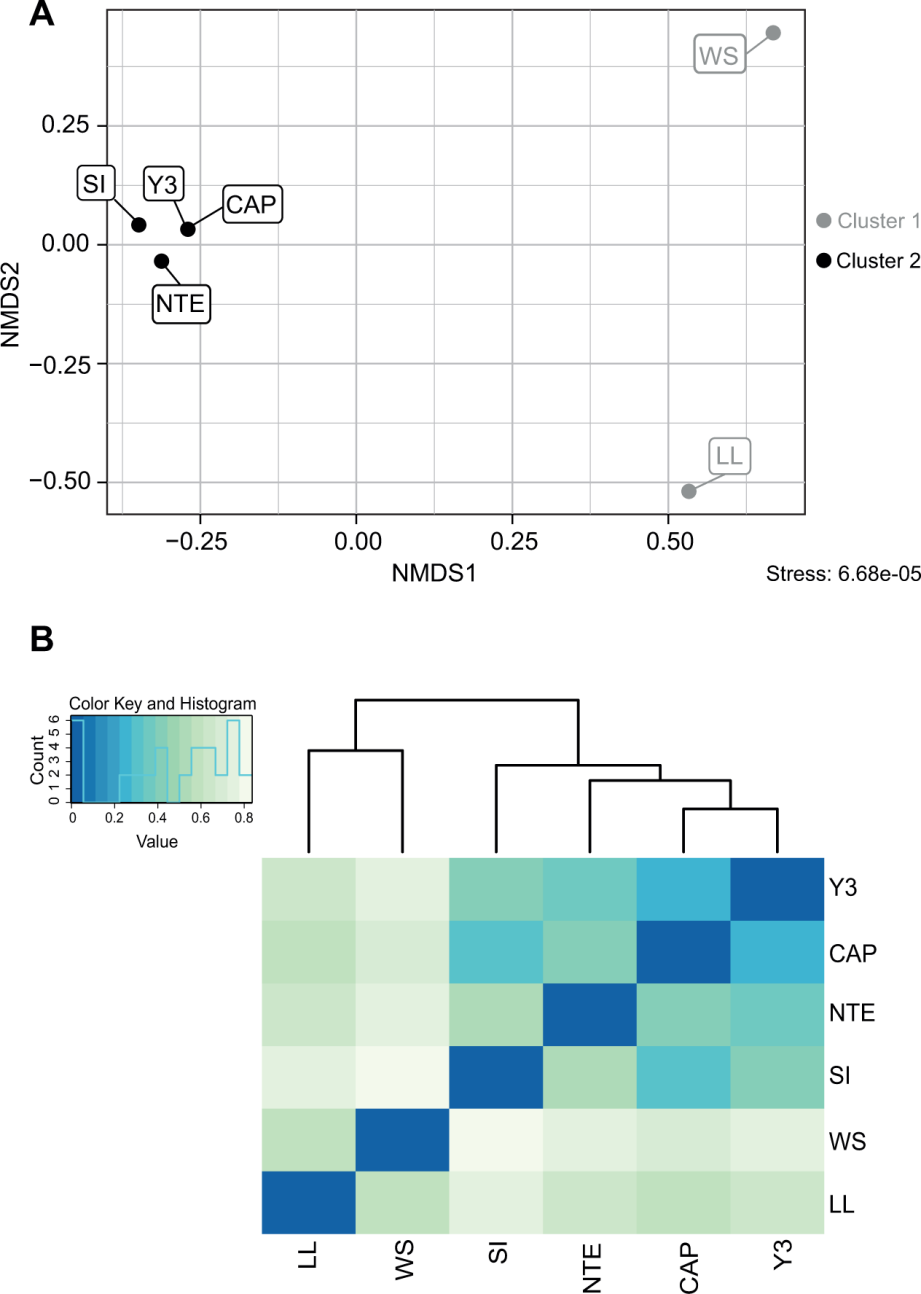
NMDS plot representing the beta diversity of the ZetaOTUs between samples using the Bray-Curtis dissimilarity index (**A**) and heatmap to visualize sample clustering (**B**). To construct this heatmap with the gplots package (57), a distance matrix between ZetaOTUs was calculated with the vegan package using the Bray-Curtis dissimilarity index.

Among the 5,593 bacterial ASVs, 155 were identified as belonging to the Zetaproteobacteria class. These bacteria were further classified into 23 different ZetaOTUs, among which two belonged to novel ZetaOTUs, i.e., NewZetaOTUs 1 and 2. Regarding the alpha diversity of the ZetaOTUs (Fig. 4A), LL, Y3, and NTE presented a higher alpha diversity (>1.5) than CAP, SI, and WS. Figure 4B shows that WS was the site with the highest relative abundance of Zetaproteobacteria (accounting for 44.95% of the bacterial community), as determined by qPCR (Table S2). Even though the relative abundances determined by qPCR and metabarcoding were consistent (Fig. 4B), qPCR provides a more reliable quantification due to the use of Zetaproteobacterial-specific 16S rRNA gene primer couples and to the absence of a bioinformatic treatment that might have eliminated certain putative Zetaproteobacterial sequences. WS was followed by LL (33.21%), SI (28.68%), Y3 (25.07%), CAP (21.55%), and finally NTE (11.69%). Interestingly, two of the iron-rich microbial mats presenting high relative abundances of Zetaproteobacteria (WS and SI) were the ones in which their communities were less diversified. The NMDS plot (Fig. 5A) revealed, according to the NMDS1 axis, a clearly defined distribution of the samples in two different clusters regarding the diversity of the ZetaOTUs. Cluster 1 grouped LL and WS, and cluster 2 included CAP, Y3, NTE, and SI. This was confirmed by the heatmap (Fig. 5B) and the PERMANOVA, which yielded a *P*-value of 0.001 between samples of different clusters.

### Zetaproteobacterial core microbiome of the LSHF

**Fig 6:**
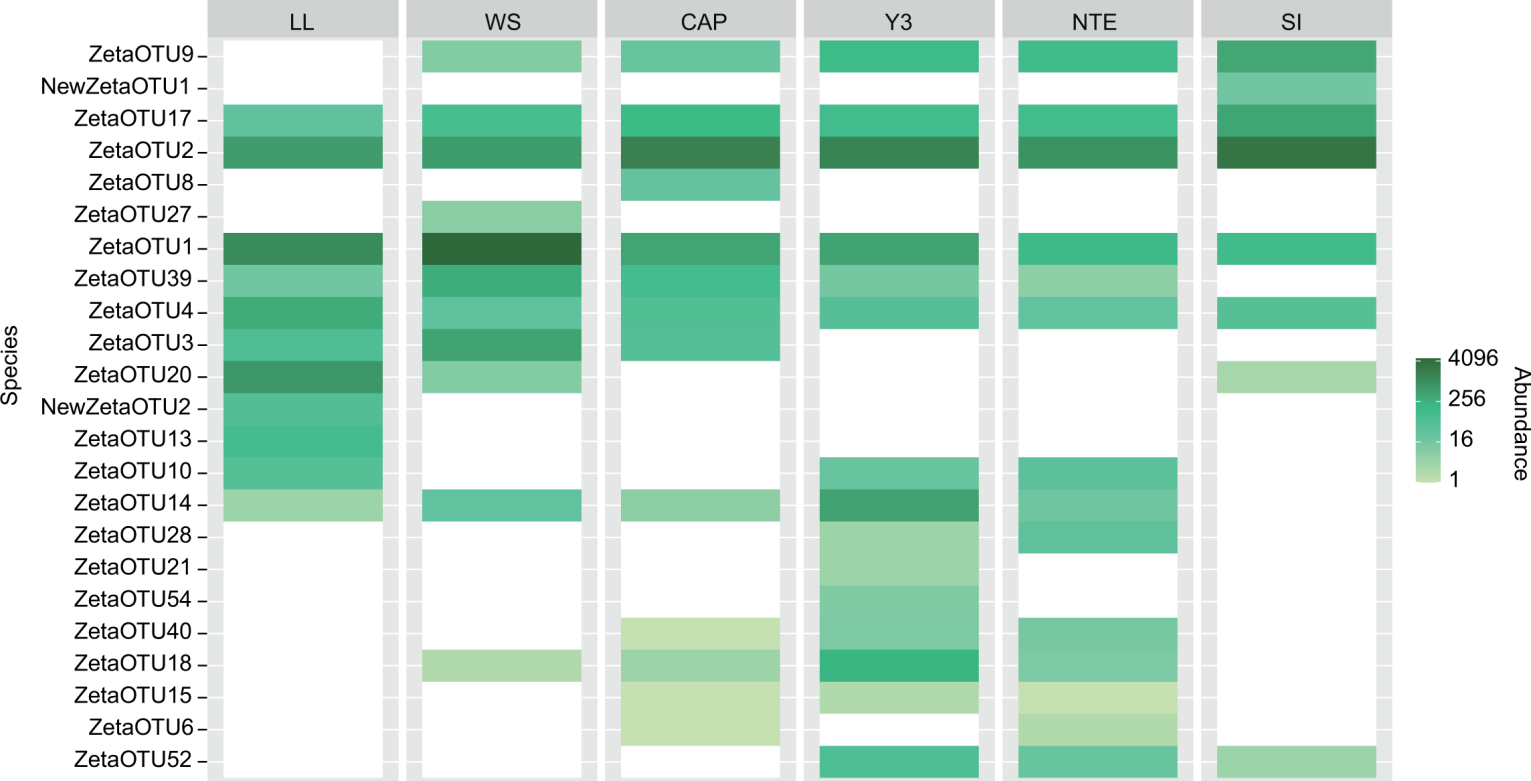
Heatmap representing the presence/absence but also the abundance of each ZetaOTU at each site. To construct this heatmap, ZetaOTUs were organized using the NMDS ordination and the Bray-Curtis dissimilarity index.

A heatmap representing the abundance of each ZetaOTU at each site (Fig. 6) showed that four different ZetaOTUs were present (>0.1% of abundance) in all the samples (ZetaOTUs 1, 2, 4, and 17), representing together between 67.5% and 91.5% of the complete Zetaproteobacterial communities in each sample. These ZetaOTUs were thus defined as the Zetaproteobacterial core microbiome of iron-rich microbial mats from the LSHF, with ZetaOTUs 1 and 2 presenting the highest abundances (Table S5). Our results show that ZetaOTUs 1 and 4, which have been mostly described in the Pacific Ocean (25, 28, 29), are cosmopolitan at the LSHF, revealing that they can be present and quite abundant in the Atlantic Ocean. The abundances of the ZetaOTUs of the Zetaproteobacterial core microbiome allowed us to differentiate the clusters defined above (Fig. 6; Table S5). In cluster 1, the lower abundance of ZetaOTUs 2 and 17, together with the higher abundance of ZetaOTU 1 compared to cluster 2, indicated that the mats developing at these two sites, LL and WS, are more similar between them than with those from cluster 2.

### Shared and distinctive characteristics of clusters

Besides the Zetaproteobacterial core microbiome, these two clusters contained other common ZetaOTUs, ZetaOTUs 9, 10, 14, 18, and 39 (Fig. 6), which were present in at least half of the samples in each cluster. ZetaOTUs 3 and 20 were present in both samples from cluster 1 and in only one sample (CAP and SI, respectively) from cluster 2.

Within cluster 1, ZetaOTU 3 presented higher abundances at WS than at LL, as opposed to ZetaOTU 20. NewZetaOTU 2 and ZetaOTU 13 were exclusive to LL, while ZetaOTU 27 was only present at WS. NewZetaOTU 2 and ZetaOTU 20 formed a monophyletic group in our phylogenetic tree (Fig. S4), indicating that they share a common ancestor and could probably present similar characteristics.

Cluster 2 contained a group of specific ZetaOTUs, 28, 40, 15, 6, and 52, present in at least two out of the four mats comprising this cluster (although some in very low abundances). NTE was the only site where all cluster 2-specific ZetaOTUs were detected. This site was characterized by a high abundance of ZetaOTUs 9 and 10. ZetaOTU 10 has been described at Lōʻihi (4, 6, 25) and at the Mariana arc and back-arc (referred to as Mariana throughout the text) (28), but this is the first time that it is reported in iron-rich mats from the MAR. CAP was characterized by the presence of ZetaOTU 8, which was only present in this mat, and of ZetaOTU 3. Both ZetaOTUs have been described at the Pacific (28) and at the AMOR (31), but this is the first time that they are described at the MAR. Besides these two ZetaOTUs, its Zetaproteobacterial communities remained very similar to those at other sites in cluster 2. Y3 was characterized by the highest abundances of ZetaOTUs 14 and 18 and by the presence of ZetaOTUs 21 and 54 only at this site. Finally, the Zetaproteobacterial community of SI was characterized by NewZetaOTU 1 and ZetaOTU 9 and lacked all the cluster 2-specific ZetaOTUs except for ZetaOTU 52. NewZetaOTU 1, which formed a monophyletic group with ZetaOTU 2 (Fig. S4), was exclusive from this site.

### Iron oxide morphologies

**Fig 7:**
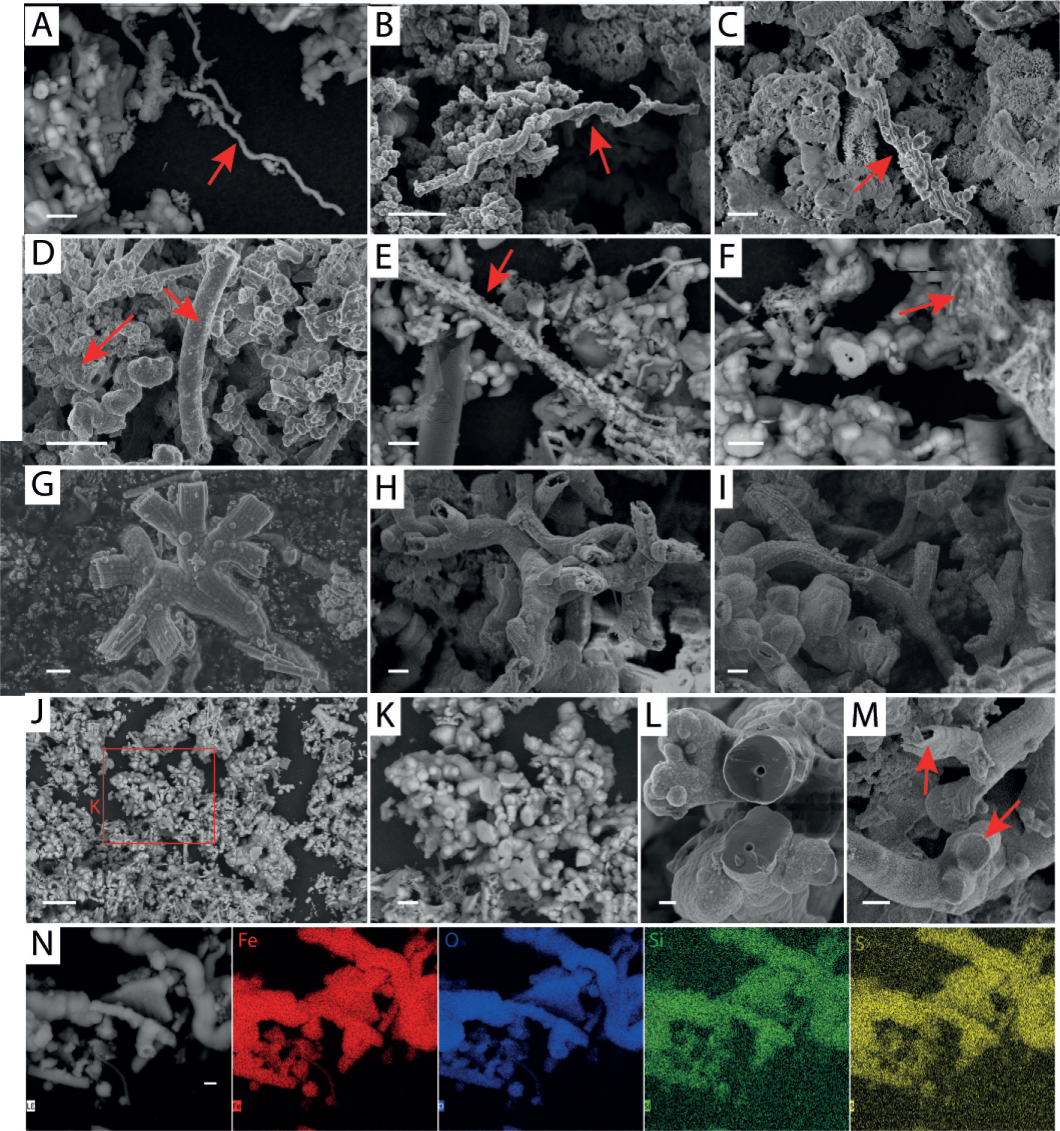
Classification of iron oxide morphologies used in our study, observed by SEM with either backscattered electron (**A, E, F, J, and K**) or secondary electron detectors (**B to D, G to I, and L to N**). Stalks (**A to C**) observed on WS 2021 (**A**), Y3 2020 (**B**), and NTE 2021 (**C**). Sheath (**D**) observed on CAP 2021. Amorphous oxides (**E**) observed on WS 2021. Nest (**F**) observed on WS 2021. Y-structures (**G to I**) observed on LL 2021 (**G**), and LL 2020 (**H and I**). Corals (**J to N**) observed on WS 2021 (**J to L**), LL 2020 (**M**), and WS 2020 (**N**). The iron oxides look heavily mineralized. View of panel K corresponds to the red rectangle in panel J. In panel **M**, note the hollow core and the individual filaments visible on the Y-structure (top) compared to the full core and smooth structure of the coral (bottom). Scale bar: 1 µm (**C, G to I, and L to N**), 5 µm (**A, B, D to F, and K**), or 20 µm (**J**).

We identified and counted five already described iron oxide morphologies within our samples: stalks, sheaths, amorphous oxides, nests, and Y-structures (22, 24, 58) (Fig. 7). Stalks are generally twisted and can comprise either an individual (Fig. 7A) or several filaments (Fig. 7B and C). Sheaths are straight and typically empty (Fig. 7D) (24). Amorphous oxides are usually formed by the abiotic oxidation of iron, but they can also be produced by Zetaproteobacteria (59). They are small, have a round shape, and are usually found in association with other morphologies, such as sheaths or stalks (Fig. 7E). Nests are aggregates of Fe hydroxide fibers (Fig. 7F), and Y-structures are defined as short hollow tubes that are formed by multiple individual Fe oxide fibrils (Fig. 7G, H, I, and M). In addition, we defined a new morphology that we named “corals.” Like Y-structures, corals present bifurcations, but they present different levels of mineralization, with a smooth surface and no visible individual fibrils (Fig. 7J through N). They can be threefold the diameter of Y-structures (3 µm vs 1 µm), and when they are sectioned, a hole can be observed in their center (Fig. 7K through N). EDS analysis evidences the co-localized occurrence of iron and oxygen (Fig. 7N), with additional minor elements, such as sulfur or silicon. Such elemental composition is identical to that of iron oxides presenting other morphologies (amorphous iron oxides and Y-structures; Fig. S5).

### Differences in abundance between sites

**Fig 8:**
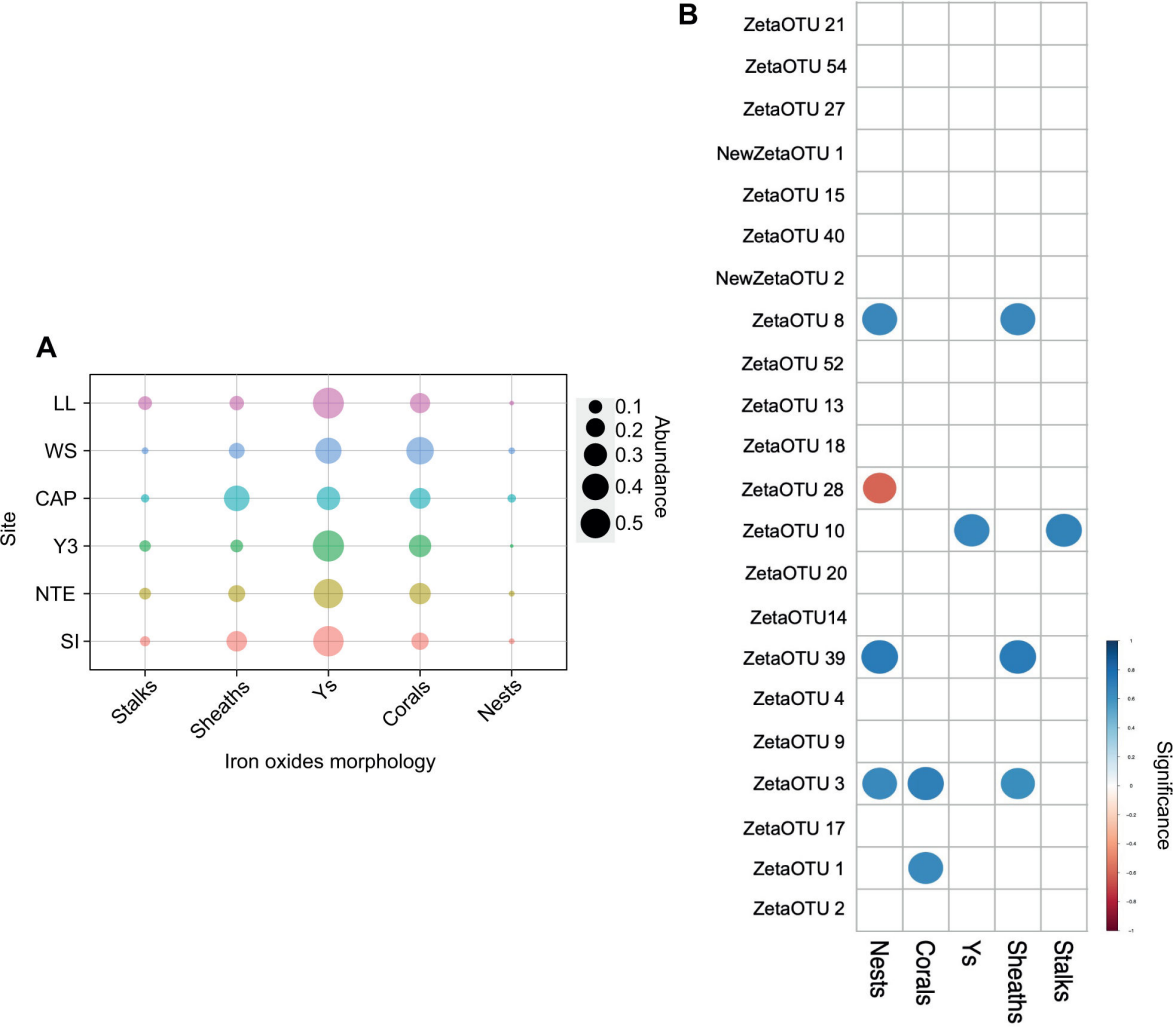
Bubble plot representing the average abundance of each iron oxide morphology at each site (Table S3) (A) and correlation matrix representing the Spearman rank correlations between the summed iron oxide morphology counts and the ZetaOTUs present at each site in the 2020 and 2021 samples (B). The Spearman rank correlation was calculated using the stats package (47), and significant correlations with a threshold of significance set to *P* < 0.05 were plotted in dark blue in a correlation matrix using the corrplot package (60).

Systematic analysis of SEM pictures revealed notable differences between the iron oxide abundances at each site (Fig. 8A; Table S6). Amorphous oxides, studied in a qualitative way, were highly abundant at LL, CAP, and Y3, presented lower abundances at WS, and were scarce at NTE and SI. A bubble plot (Fig. 8A) indicated that clusters 1 and 2 cannot be distinguished according to iron oxide morphologies and that around 30%–50% of the iron oxides corresponded to Y-structures in all the samples. These were followed by corals (20%–40%), sheaths (10%–20%), and stalks (3%–10%). Nests represented a very low proportion of the iron oxides (1%–4%). LL, Y3, and NTE mats were dominated by Y-structures, followed by corals, while the opposite was observed at WS. At CAP, iron oxides were predominantly sheaths, and SI was dominated by Y-structures, followed by sheaths.

### Relationship between iron oxide morphology and ZetaOTUs

The results of the Spearman correlation (Fig. 8B; Fig. S6; Table S7) between the abundances of specific iron oxide morphologies and ZetaOTUs showed that significant positive and negative correlations can be drawn. The abundance of stalks was significantly correlated with that of ZetaOTU 10. This ZetaOTU was highly abundant at LL, Y3, and NTE, where stalks presented the highest abundance. The abundance of sheaths was significantly correlated with that of ZetaOTUs 3, 8, and 39. These ZetaOTUs were only present all at once at CAP, the only iron-rich microbial mat in which sheaths dominated. ZetaOTU 3 was also present at LL and WS, while ZetaOTU 39 was present at all sites except for SI. The abundance of Y-structure was significantly correlated with the abundance of ZetaOTU 10, which was identified at LL, NTE, and Y3, where Y-structures were the dominant morphology. The abundance of corals was significantly correlated with that of ZetaOTUs 1 and 3, which were both more abundant at WS than at the other sites, where the mats presented the highest proportion of corals. Finally, the abundance of nests was significantly correlated with that of ZetaOTUs 3, 8, and 39, the same ZetaOTUs that presented a link with sheaths. It should be noted that the abundance of nests presented the only negative significant correlation with that of ZetaOTU 28.

## DISCUSSION

### Main bacterial players in iron-rich mats from the LSHF

Zetaproteobacteria are autotrophic bacteria and are considered to be one of the primary producers of marine iron-rich microbial mats (61). Here, they represented between 11.69% and 44.95% of the bacterial communities (Fig. 3; Fig. 4B; Table S2), which is in accordance with what has been found in previous studies of iron-rich mats from other deep-sea hydrothermal environments (28, 30, 31). Zetaproteobacteria was the most abundant class within the Proteobacteria phylum at WS, LL, SI, and Y3, accounting therefore for a large proportion of primary production, as opposed to CAP and NTE, where they did not dominate (32). Members of the Patescibacteria and Bacteroidota phyla also presented high abundances in all the samples (Fig. 3; Table S4). As previously proposed for both iron-rich microbial mats (32) and other deep-sea hydrothermal vent biofilms (62), the heterotrophic members of these phyla could use the organic polymers produced by the primary producers for growth. Indeed, their presence confirms that the autotrophic primary producers of these communities are able to recruit heterotrophic microorganisms to develop the mats. Besides, we highlight a clear difference regarding sulfur metabolism between the samples belonging to the different clusters defined for the Zetaproteobacterial communities. In cluster 2, both sulfur-oxidizing and sulfate-reducing bacteria, mainly belonging to the Campylobacterota and Desulfobacterota phyla, were present and quite abundant within the bacterial communities, indicating the presence of an active sulfur cycle in this cluster. Conversely, this cycle seems to be less active in cluster 1, where these groups were either absent or presented very low abundances, in accordance with the absence or low hydrothermal influence at LL and WS, respectively. Within cluster 2, we also observed a higher abundance of bacterial ASVs belonging to the *Geopsychrobacter* genus, known as iron-reducing bacteria, especially at NTE and SI. The iron-rich mats at these two sites are the only ones to develop close or directly on top of steel chains, an external source of Fe(III) for these bacteria. Finally, we observed a particularity at LL, which presented the highest abundances of the Chloroflexi and Planctomycetota phyla. Previous studies of the microbial diversity present on seafloor basalts have already shown the predominance of these two phyla in such habitats (63–67).

### Shared characteristics between clusters highlight the essential role of some ZetaOTUs

Focusing on Zetaproteobacteria, our analysis identifies for the first time the Zetaproteobacterial core microbiome of iron-rich microbial mats from the LSHF, comprising ZetaOTUs 1, 2, 4, and 17 (Fig. 6). These results corroborate that ZetaOTU 2, characterized as globally cosmopolitan (4, 25, 31), and ZetaOTU 17, identified both at the Pacific and at the other sites of the MAR (Rainbow, TAG, or SnakePit) (25, 29), are present in iron-rich mats from the LSHF. We also show the importance of ZetaOTUs 1 and 4 at the MAR. ZetaOTU 1 is one of the most prevalent ZetaOTUs in the Pacific Ocean (28, 29), and it has been suggested that it may only exist there (25). Such assumption appears inconsistent with its identification at the Troll Wall Vent Field in the AMOR (31) and in our iron-rich microbial mats from the LSHF at the MAR. ZetaOTU 4 is cataloged as cosmopolitan across the Pacific (4, 28, 29) and presents very low abundances at other sites of the MAR (23, 25) or the AMOR (30). Our findings show that ZetaOTUs 1 and 4 are cosmopolitan at the LSHF, revealing their previously neglected importance in the Atlantic Ocean. The assessment of the Zetaproteobacterial core microbiome allowed us to identify the common ZetaOTUs among all the samples and therefore those that seem to be crucial for the development of the iron-rich microbial mat communities at the LSHF.

All the other common ZetaOTUs between clusters (ZetaOTUs 9, 10, 14, 18, and 39) have previously been detected at different hydrothermal sites across the globe (23, 25, 28, 30, 31, 68). ZetaOTU 9 is particularly known for playing a crucial role in subsurface seawater (54, 69), mineral weathering (8, 59), and metal corrosion environments (5, 8, 70). The most prevalent ASVs affiliated with this ZetaOTU presented >97% sequence similarity to *Ghiorsea bivora*, which can use both dFe and molecular hydrogen (H_2_) as sole electron donors for growth (11). The oxidation of H_2_ is more energetically favorable than that of dFe (67), giving a competitive advantage to members of ZetaOTU 9 (11, 18) to develop in hydrothermal environments enriched in H_2_ compared to seawater. This explains the presence of ZetaOTU 9 at all sites of cluster 2 and at WS, which are associated with active hydrothermal sites, and its absence at LL, where no hydrothermal diffuse outflow has been observed. Moreover, the mats developing at NTE and SI develop really close or directly on waste steel chains and ballast from previous HOV descents. These anthropogenically introduced elements could release H_2_ due to their reaction with surrounding seawater (11, 71–73), explaining the highest abundances of ZetaOTU 9 at these sites.

### Differences between clusters reveal the influence of substratum on the Zetaproteobacterial communities

Drawing firm conclusions about the relationships between environmental conditions and Zetaproteobacterial diversity patterns using a limited number of samples for each environmental condition remains a challenge. Nonetheless, here, we investigate and discuss how different substrata presenting different permeability levels host iron-rich mats with distinct Zetaproteobacterial communities.

#### Cluster 1

Iron-rich microbial mats from LL grow on a basaltic substratum, which has low permeability and where no hydrothermal diffuse outflow has been observed. WS mats develop on massive sulfide hydrothermal deposits that form rugged terrain and can hinder the outflow of diffuse fluids (33). The similarities between the Zetaproteobacterial communities of WS and LL microbial mats, which in turn develop on substrata with similar permeabilities, could indicate a link between the Zetaproteobacterial diversity and the substratum. However, differences between the Zetaproteobacterial communities of LL and WS could still be observed both in the NMDS (Fig. 5A) and the heatmap (Fig. 6), probably linked to the different mineralogical compositions of their substrata (basalt for LL vs polymetallic sulfide deposits at WS) and to the presence of low hydrothermal fluid flux at WS.

Cluster 1 was characterized by the presence of ZetaOTU 3, which is cosmopolitan in the Pacific (28, 29, 74) and has also been described at the AMOR (31). The ASVs affiliated with ZetaOTUs 13, 20, 27, and 39, which were exclusive or presented their highest abundances in this cluster, had >97% sequence identity with uncultured bacterium clones detected in Mariana (McAllister, S.M. and Chan, C.S., Accession Nos. MK048939.1, MK048935.1, and MK048685.1, GenBank - NCBI) (27, 68). This, together with the higher abundance of ZetaOTUs 1 and 4, mostly described in the Pacific, revealed that a high proportion of the ZetaOTUs found in LL and WS have previously been detected in iron-rich microbial mats from the Pacific Ocean.

At LL, the ASVs identified as NewZetaOTU 2 and ZetaOTU 20 presented between 96.7% and 99.3% sequence similarity with an uncultured bacterium clone detected at the Snail site in Mariana (27) (Accession No. EU574668.1, GenBank - NCBI). ZetaOTU 20 had already been detected at Snail (28), which hosts an iron-rich microbial mat presenting a black outer surface, like LL (Fig. 2). Even though this black deposit on the mat’s surface has not been analyzed, previous studies suggested that it is composed of manganese (Mn) oxides (28), such as those found at the Juan de Fuca Ridge (75). The presence of ZetaOTU 20 at both LL and Snail suggests that this ZetaOTU could be involved in the Mn biogeochemical cycle, either as a potential Fe and Mn oxidizer, like the freshwater iron oxidizer *Leptothrix discophora* (76), or as a potential recruiter of other Mn oxidizers present in the mats and in the fluids surrounding them, as described in multiple hydrothermal environments (77–79).

#### Cluster 2

Despite specificities, the Zetaproteobacterial communities in cluster 2 resembled one another. CAP, NTE, and SI iron mats form on hydrothermally cemented breccia named hydrothermal slab (33, 36, 80). Within these types of substrata, active sites usually present a focused high-temperature discharge zone and diffuse fluids within the surrounding areas. The iron-rich microbial mats from these sites are those that develop further away from the active sites (Fig. 2) but in close relation with the fissures releasing diffuse fluids. Y3 mats develop on hydrothermal gravity deposits, a highly complex and heterogeneous substratum formed by accumulated hydrothermal rock blocks, which are associated with an extended network of cracks, directly influencing the mats (80). The similarities in the substratum between CAP, NTE, and SI and the presence of diffuse outflow at these three sites and at Y3 are reflected in the Zetaproteobacterial diversity present in them.

The groups of ZetaOTUs 28, 40, 15, 6, and 52 were specific to cluster 2. Among them, ZetaOTUs 28 and 15 are abundant in iron-rich microbial mats from the MAR (23, 25), and ZetaOTUs 6 and 15 have been reported as some of the most abundant ZetaOTUs at the AMOR (31). Moreover, ZetaOTUs 2 and 17 from the Zetaproteobacterial core microbiome presented higher abundances in this cluster. Overall, these results showed that a large proportion of cluster 2 ZetaOTUs have mainly, but not exclusively, been described at other vent sites along the MAR or the AMOR, suggesting that cluster 2 mats are more similar to them than those of cluster 1.

Within cluster 2, there were also some differences between the mats, as evidenced by the presence or high abundance of some ZetaOTUs (Fig. 6). CAP is a particular site located near a deep fault with associated end-member hydrothermal fluids presenting dFe concentrations 4–16 times higher than those of the other sites (38). Besides the presence of ZetaOTUs 3 and 8, the CAP Zetaproteobacterial communities remained very similar to those of the other sites from cluster 2. This indicates that the nutrient supply provided by hydrothermal fluids does not appear to have any specific effect on the Zetaproteobacterial diversity of the mats, in contrast to what has been described for their entire microbial communities (32). Y3 is located at the base of a less deep, steep scarp fault, where hydrothermal fluid, besides experiencing high compositional variations through time (81), arrives more directly. Y3 was one of the most diversified mats in terms of ZetaOTUs (Fig. 4A). At this site, the most characteristic ZetaOTUs have previously been detected in brackish waters (9), in shallow hydrothermal Fe-oxyhydroxide deposits in Nagahama Bay (Japan) (82, 83), and in terrestrial carbonic iron-rich springs (18, 84–86). The presence of such differing ZetaOTUs at Y3 could indicate that its mats harbor niches that can potentially be found in very distinctive habitats, which could be explained by the complexity and heterogeneity of the substratum at this location. SI presented the lowest alpha diversity index (Fig. 4A), as it only harbored the ZetaOTUs from the Zetaproteobacterial core microbiome, NewZetaOTU 1 and ZetaOTUs 9, 20, and 52. The only ASV corresponding to NewZetaOTU 1, which was very closely related phylogenetically to ZetaOTU 2 (Fig. S4), presented 98.4% sequence similarity with an uncultured Zetaproteobacterial bacterium clone detected in an iron-rich microbial mat in Mariana (McAllister, S.M. and Chan, C.S, Accession No. MK048928.1, GenBank - NCBI). This site is clearly unique, as iron-rich microbial mats develop on top of steel chains, ropes, and ballasts, which could be linked to the high abundance of ZetaOTU 9. SI is therefore the best illustration of the importance of the Zetaproteobacterial core microbiome for the formation of iron-rich mats at the LSHF.

### Iron oxide morphology could be taxonomically and/or environmentally driven

The bulk of iron-rich microbial mats have typically been described as being mostly composed of stalks or sheaths (24). Twisted stalks are produced not only by Zetaproteobacteria in the marine environment but also by freshwater Betaproteobacteria (*Gallionella ferruginea*) (87). Sheath formers have not yet been isolated among the Zetaproteobacteria, but they have been well studied in freshwater systems (*Leptothrix ochracea*) (88). Within our samples, only CAP mats were dominated by sheaths (Fig. 8A). On the contrary, the predominant iron oxide morphologies at LL, WS, Y3, NTE, and SI were Y-structures and corals, a new structure described here for the first time. Y-structures were confirmed to be formed by Zetaproteobacteria (89, 90) and are now considered to be one of the predominant iron oxide morphologies produced by this bacterial class, together with stalks and sheaths. Regarding corals, our analyses reveal the need to isolate a Zetaproteobacterial strain producing this iron oxide morphology to gain further evidence about their biological origin.

In our samples, the abundances of stalks and Y-structures were both significantly correlated with that of ZetaOTU 10, while the abundances of sheaths and nests both presented significant correlations with those of ZetaOTUs 3, 8, and 39 (Fig. 8B; Fig. S6; Table S7). Two hypotheses could explain these observations: either different species within one ZetaOTU are specialized in the production of an iron oxide morphology or all species of a ZetaOTU are able to produce different oxide morphologies depending on the environmental conditions. For instance, Y-structures are usually shorter than stalks. It has been proposed that the microorganisms involved in their formation stop the mineralization earlier due to stress induced by different environmental variations, forcing them to colonize another niche (24). Besides these observations, ZetaOTUs 1 and 3, both significatively associated with corals, formed a monophyletic group (Fig. S4), which could indicate that coral formation is a trait linked to phylogeny. Finally, the ZetaOTUs that exhibited a significant positive correlation with nest abundance (i.e., ZetaOTUs 3, 8, and 39) were either absent or presented very low abundances at the sites where ZetaOTU 28 was present, which could explain the negative significant correlation between the abundance of nests and that of ZetaOTU 28.

Such picture of the relationship between Zetaproteobacterial diversity and iron oxide morphology would benefit from a precise spatial sampling to gain additional knowledge of the function and niche of iron oxides. Nonetheless, our results argue that iron oxide morphologies can be produced by specific ZetaOTUs *in situ* within natural mats, as already demonstrated *in vitro* with cultured Zetaproteobacterial representatives (18). Our analyses suggest that iron oxide morphology production could be both taxonomically and/or environmentally driven. Further evidence could be obtained by examining the iron oxide morphologies produced by Zetaproteobacteria enrichments from mats dominated by a specific iron oxide morphology.

## Data Availability

The data sets generated and analyzed during the current study are publicly available. The data from CAP and NTE 17-20 samples have been already published and can be found here: NCBI, PRJNA798257, the accession numbers for the BioSamples are SAMN25059546 - SAMN25059555. All the other data can be found here: NCBI, PRJNA798257, the accession numbers for the BioSamples are SAMN35005788 - SAMN35005797.

## SUPPLEMENTAL MATERIAL

Supplementary material aem.02041-23-s0001.docx Supplementary document containing supplementary materials and methods, supplementary figures, and supplementary tables.

ASM does not own the copyrights to Supplemental Material that may be linked to, or accessed through, an article. The authors have granted ASM a non-exclusive, world-wide license to publish the Supplemental Material files. Please contact the corresponding author directly for reuse.
