# Supplementary material for: Linking Zetaproteobacterial diversity and substratum type in iron-rich microbial mats from the Lucky Strike hydrothermal field (EMSO-Azores observatory)
Source: Appl Environ Microbiol. 2024 Jan 9;90(2):e02041-23. doi: 10.1128/aem.02041-23 (PMC10880625; doi:10.1128/aem.02041-23)
Supplement: Supplementary material — Supplementary document containing supplementary materials and methods, supplementary figures, and supplementary tables. [file aem.02041-23-s0001.docx]

**SUPPLEMENTARY MATERIAL**

**SUPPLEMENTARY MATERIAL AND METHODS**

**Site and sample description**

At LL, the substratum consists of fresh, pillow and lobate lavas and sheet basaltic flows, building pillow mounds of up to 2 m high (1, 2). At WS, the oxidized massive sulphide blocks are composed of copper and iron hydroxides, with pyrite and chalcopyrite (3). The mats are found at 6 m to the west of the Sintra active edifice, with a ~5-m high spire and three other chimneys venting high-temperature fluids up to 212ºC (3, 4). At CAP, microbial mats develop on a 10-m high sulphide mound. They are located at 32 m from the active vents, characterized by ~15-m high candelabra-shape chimneys discharging fluids at temperatures of up to 324°C (5, 6). Y3 mats develop ~5 m away from the Y3 active site, which exhibits a pedestal morphology on which several chimneys discharge hydrothermal fluids (up to 324ºC) (7). At NTE, iron-rich microbial mats develop 21 m to the north of the main Tour Eiffel’s edifice. This active vent is located at the south-east side of the fossil lava lake and consists of a ~18-m high tower-like main edifice, with 4 or 5 chimneys venting hot fluids up to 326°C, surrounded at its base by a large network of fissures and cracks where diffuse hydrothermal fluids discharge at temperatures of up to 100°C (8, 9). The mats from SI develop 80 m to the south of Isabel site, located at the south-east side of the lava lake, ~160 m away from Tour Eiffel. It consists of three high-temperature vents, in addition to two <1-m high active chimneys at the base of the edifice and diffuse venting on its flank (4).

**DNA extraction and 16S rRNA gene sequences analyses**

Primers 16S rRNA gene for Bacteria:

341F (5’-CCTACGGGNGGCWGCAG)

785R (5’-GACTACHVGGGTATCTAATCC)

The PCR program consisted of a 5-minute denaturation step at 95°C, followed by 30-35 cycles of a 30-second step at 95°C, a 40-second step at 53°C and a 1-minute step at 72°C, and a final 10-minute elongation step at 72°C.

**Diversity analyses**

The Shannon diversity index was calculated to represent the ZetaOTUs alpha diversity (Figure 3A). Non-Metric Multidimensional Scaling (NMDS) analyses were performed to represent the bacterial and Zetaproteobacterial beta diversity between all samples from the six sites, using all ASVs corresponding to Bacteria or to the Zetaproteobacteria class (Figure S3A and S3B, respectively). NMDS analyses were also performed to represent the ZetaOTUs beta diversity between sites (Figure 4A). The ordinations were produced using distance matrices calculated according to the Bray-Curtis dissimilarity index, as the number of null values between samples does not affect it. A Permutational Multivariate Analysis of Variance (PERMANOVA, from the vegan package (10)) using distance matrices was performed to assess whether the differences observed in the NMDS between all samples from the six sites were significant (Figure S3), and to assess if the differences in ZetaOTUs between clusters were significant, considering all samples from the six sites as well. The threshold of significance was set at a p-value of 0.05.

**qPCR**

Primers 16S rRNA gene for Bacteria:

Bac1369F (5′-CGGTGAATACGTTCYCGG-3′)

Prok1492R (5′-GGWT ACCTTGTTACGACTT-3′)

Primers 16S rRNA gene for Zetaproteobacteria:

Zeta672F (5’-CGGAATTCCGTGTGTAGCAGT-3’)

Zeta837R (5’-GCCACWGYAGGGGTCGATACC-3’)

Each qPCR reaction mixture contained 7.5 μl of SsoAdvanced Universal SYBR^®^ Green SuperMix (Bio-Rad), primers at a final concentration of 500 nM, DNase-free water and 2 μl of DNA in a final volume of 13 μl. Each reaction was performed in duplicate, and each plate also contained a standard curve and a negative control sample. The standard curves were obtained by serial dilution of genomic DNA from a pure culture of *Ghiorsea bivora*. The PCR program for Bacteria and Zetaproteobacteria consisted of a 2-minute initial denaturation step at 98°C, followed by 45 successive cycles of a 5-second denaturation step at 98°C and an hybridization step of 10 seconds at 60°C. Finally, a 10-second denaturation step at 95°C was performed. Melting curves were generated at the end of each qPCR from 65 to 95°C (in 5°C steps) for both Bacteria and Zetaproteobacteria.

**Scanning Electron Microscopy**

On the Zeiss SEM, low ow magnification pictures (<x1000) were taken at an accelerating potential of 15 keV under variable pressure and high magnification pictures (>x1000) at 3 kV under high vacuum. Samples were placed uncoated on a carbon tape with a drop of ethanol to avoid precipitation of salt. A 4 nm-gold coating was occasionally applied to inhibit charging.

On the TESCAN SEM, analysis was performed at an accelerating potential of 15keV under high vacuum and a WD of 10 mm. Samples were carbon-coated (15 nm) prior to analysis.

**SUPPLEMENTARY FIGURES**

**Figure S1.** Representation of the selection of the same three 900x600 pixels areas from the highest quality x400 image of each sample in backscattering electron mode. The multi-point tool was used for counting each iron oxide morphology.

**Figure S2.** Rarefaction curves for Bacteria. Sample names consist on the name of the site followed by the year of sampling.

**Figure S3.** NMDS plot representing the bacterial **(A)** and Zetaproteobacterial **(B)** beta diversity.

A

B

**Figure S4.** Phylogenetic tree containing the 16S rRNA gene sequence of the ZetaOTUs found in our samples, the closest sequence of each ZetaOTU and different cultured Zeta- and Betaproteobacteria was constructed. *Acidicapsa acidiphila* was added as an outgroup to root the tree. The alignment of the sequences was performed using MUSCLE (11) and the tree was constructed with the RaxML software (12), using the maximum likelihood method and the GTR-gamma nucleotide substitution model.


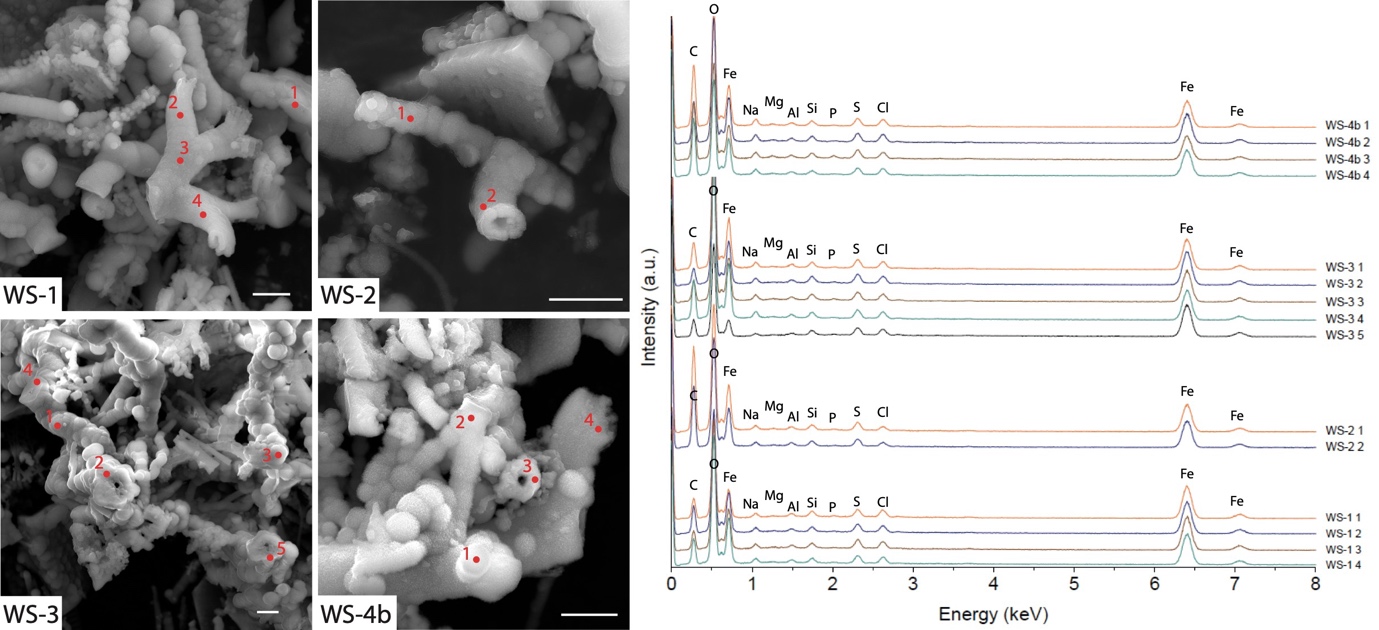


**Figure S5.** SEM pictures and EDS spectra recorded on various iron oxide morphologies of the WS 2020 sample (WS-1: Y-structure, WS-2: coral, WS-3 and WS-4b: mixing of amorphous iron oxides and corals). Scale bar = 2 µm.

**Figure S6.** Data points and regression lines for each reported significant spearman correlation between the abundance of iron oxide morphologies and that of certain ZetaOTUs, correlation coefficient (r-value) and p-value are also presented.

**SUPPLEMENTARY TABLES**

**Table S1.** Illumina MiSeq run for each sample and number of reads after each step of the DADA2 pipeline.

**Table S2.** Results from qPCR and % of Zetaproteobacteria in each sample. The average relative abundance of Zetaproteobacteria by qPCR and by 16S rRNA gene sequencing (metabarcoding) in each site are also presented.

**Table S3.** Raw counts of each iron oxide morphology from the three areas of each image. Sum and normalization by sample of the counts are also represented.

**Table S4.** Relative abundance of each bacterial phylum and Proteobacteria class determined by metabarcoding at each site (%). Only the phyla having an incidence higher than 1% are presented.

**Table S5.** Relative abundance of each ZetaOTU determined by metabarcoding at each site (%).

**Table S6.** Relative abundance of each iron oxide morphology at each site (%). The % of each iron-oxide morphology are calculated from the sum of normalized counts at each sample (Table S3). Amorphous oxides were quantified in a qualitative way.

**Table S7.** Spearman correlation coefficients (r-values) and their respective p-values between the relative abundance of each iron-oxide morphology and that of each ZetaOTU.

**SUPPLEMENTARY REFERENCES**

1. Ondréas H, Cannat M, Fouquet Y, Normand A, Sarradin PM, Sarrazin J. 2009. Recent volcanic events and the distribution of hydrothermal venting at the Lucky Strike hydrothermal field, Mid-Atlantic Ridge. Geochemistry, Geophysics, Geosystems 10.

2. Fouquet Y, Charlou J-L, Costa I, Donval J-P, Radford-Knoery J, Pelle H, Ondreas H, Lourenco N, Segonzac M, Tivey MK. 1994. A detailed study of the Lucky Strike hydrothermal site discovery of a new hydrothermal site: Menez Gwen; preliminary results of the DIVA1 cruise [5–29 May 1994]. InterRidge News 3:14–17.

3. Langmuir CL, Charlou JL, Colodner D, Corey S, Costa J, Desbruyères D, Desonie D, Emerson T, Fornari D, Fouquet Y, Humphris S, Fiala-Medioni A, Saldanha L, Sours-Page R, Thatcher M, Tivey MK, Van Dover CL, Von Damm KL, Wiese K, Wilson C. 1993. Lucky Strike-A newly discovered hydrothermal site on the Azores platform. RIDGE Events 4:3–5.

4. Barreyre T, Escartín J, Sohn RA, Cannat M, Ballu V, Crawford WC. 2014. Temporal variability and tidal modulation of hydrothermal exit-fluid temperatures at the Lucky Strike deep-sea vent field, Mid-Atlantic Ridge. J Geophys Res Solid Earth 119:2543–2566.

5. Escartin J, Barreyre T, Cannat M, Garcia R, Gracias N, Deschamps A, Salocchi A, Sarradin P-M, Ballu V. 2015. Hydrothermal activity along the slow-spreading Lucky Strike ridge segment (Mid-Atlantic Ridge): Distribution, heatflux, and geological controls. Earth Planet Sci Lett 431:173–185.

6. Chavagnac V, Leleu T, Fontaine F, Cannat M, Ceuleneer G, Castillo A. 2018. Spatial Variations in Vent Chemistry at the Lucky Strike Hydrothermal Field, Mid-Atlantic Ridge (37°N): Updates for Subseafloor Flow Geometry From the Newly Discovered Capelinhos Vent. Geochemistry, Geophysics, Geosystems 19:4444–4458.

7. Barreyre T, Escartín J, Garcia R, Cannat M, Mittelstaedt E, Prados R. 2012. Structure, temporal evolution, and heat flux estimates from the Lucky Strike deep-sea hydrothermal field derived from seafloor image mosaics. Geochemistry, Geophysics, Geosystems 13:1–29.

8. Rommevaux C, Henri P, Degboe J, Chavagnac V, Lesongeur F, Godfroy A, Boulart C, Destrigneville C, Castillo A. 2019. Prokaryote Communities at Active Chimney and In Situ Colonization Devices After a Magmatic Degassing Event (37°N MAR, EMSO-Azores Deep-Sea Observatory). Geochemistry, Geophysics, Geosystems 20:3065–3089.

9. Sarradin PM, Waeles M, Bernagout S, Le Gall C, Sarrazin J, Riso R. 2009. Speciation of dissolved copper within an active hydrothermal edifice on the Lucky Strike vent field (MAR, 37°N). Science of the Total Environment 407:869–878.

10. Oksanen J, Blanchet FG, Friendly M, Kindt R, Legendre P, McGlinn D, Minchin PR, O’Hara RB, Simpson GL, Solymos P, Stevens MHH, Szoecs E, Wagner H. 2020. Vegan: Community Ecology Package.

11. Edgar RC. 2004. MUSCLE: multiple sequence alignment with high accuracy and high throughput. Nucleic Acids Res 32:1792–1797.

12. Stamatakis A. 2014. RAxML version 8: a tool for phylogenetic analysis and post-analysis of large phylogenies. Bioinformatics 30:1312–1313.
